# Urinary prostanoids are elevated by anti-TNF and anti-IL6 receptor disease-modifying antirheumatic drugs but are not predictive of response to treatment in early rheumatoid arthritis

**DOI:** 10.1186/s13075-024-03295-9

**Published:** 2024-03-05

**Authors:** Jianyang Liu, Helena Idborg, Marina Korotkova, Kristina Lend, Ronald van Vollenhoven, Jon Lampa, Anna Rudin, Dan Nordström, Bjorn Gudbjornsson, Gerdur Gröndal, Till Uhlig, Kim Hørslev-Petersen, Merete Lund Hetland, Mikkel Østergaard, Michael Nurmohamed, Per-Johan Jakobsson

**Affiliations:** 1 Division of Rheumatology, Department of Medicine, Karolinska Institutet and Karolinska University Hospital, Solna, Stockholm, Sweden; 2 Department of Rheumatology and Amsterdam Rheumatology Center, Amsterdam University Medical Centers, Amsterdam, The Netherlands; 3 Department of Rheumatology and Inflammation Research, University of Gothenburg Sahlgrenska Academy, Gothenburg, Sweden; 4 Department of Medicine and Rheumatology, Helsinki University Hospital and University of Helsinki, Helsinki, Finland; 5 Department of Rheumatology, Landspitali University Hospital, Reykjavik, Iceland; 6 Faculty of Medicine, University of Iceland, Reykjavik, Iceland; 7 Division of Rheumatology and Research, Diakonhjemmet Hospital, Oslo, Norway; 8 University of Oslo, Oslo, Norway; 9 Danish Hospital for the Rheumatic Diseases, Sønderborg, Denmark; 10 University of Southern Denmark, Odense, Denmark; 11 Copenhagen Center for Arthritis Research (COPECARE), Center for Rheumatology and Spine Diseases, Centre for Head and Orthopaedics, Rigshospitalet, Glostrup, Denmark; 12 Department of Clinical Medicine, Faculty of Health and Medical Sciences, University of Copenhagen, Copenhagen, Denmark; 13 Amsterdam Rheumatology and Immunology Center, Reade, The Netherlands

**Keywords:** Rheumatoid arthritis, tPGEM, tPGDM, 2,3-dinor-TXB_2_, 2,3-dinor-6-keto-PGF_1a_, LTE_4_, 12-HETE

## Abstract

**Background:**

Disease-modifying antirheumatic drugs (DMARDs) are widely used for treating rheumatoid arthritis (RA). However, there are no established biomarkers to predict a patient’s response to these therapies. Prostanoids, encompassing prostaglandins, prostacyclins, and thromboxanes, are potent lipid mediators implicated in RA progression. Nevertheless, the influence of DMARDs on prostanoid biosynthesis in RA patients remains poorly understood. This study aims to assess the impact of various DMARDs on urinary prostanoids levels and to explore whether urinary prostanoid profiles correlate with disease activity or response to therapy.

**Methods:**

This study included 152 Swedish female patients with early RA, all rheumatoid factor (RF) positive, enrolled in the NORD-STAR trial (registration number: NCT01491815). Participants were randomized into four therapeutic regimes: methotrexate (MTX) combined with (i) prednisolone (arm ACT), (ii) TNF-α blocker certolizumab pegol (arm CZP), (iii) CTLA-4Ig abatacept (arm ABA), or (iv) IL-6R blocker tocilizumab (arm TCZ). Urine samples, collected before start of treatment and at 24 weeks post-treatment, were analyzed for tetranor-prostaglandin E metabolite (tPGEM), tetranor-prostaglandin D metabolite (tPGDM), 2,3-dinor thromboxane B_2_ (TXBM), 2,3-dinor-6-keto prostaglandin F_1a_ (PGIM), leukotriene E_4_ (LTE_4_) and 12-hydroxyeicosatetraenoic acid (12-HETE) using liquid chromatography–mass spectrometry (LC–MS). Generalized estimating equation (GEE) models were used to analyze the change in urinary eicosanoids and their correlations to clinical outcomes.

**Results:**

Patients receiving MTX combined with CZP or TCZ exhibited significant elevations in urinary tPGEM and TXBM levels after 24 weeks of treatment. Other eicosanoids did not show significant alterations in response to any treatment. Baseline urinary eicosanoid levels did not correlate with baseline clinical disease activity index (CDAI) levels, nor with changes in CDAI from baseline to week 24. Their levels were also similar between patients who achieved CDAI remission and those with active disease at week 24.

**Conclusions:**

Treatment with anti-TNF or anti-IL6R agents in early RA patients leads to an increased systemic production of proinflammatory and prothrombotic prostanoids. However, urinary eicosanoid levels do not appear to be predictive of the response to DMARDs therapy.

**Supplementary Information:**

The online version contains supplementary material available at 10.1186/s13075-024-03295-9.

## Background

Rheumatoid arthritis (RA) is an autoimmune disease affecting around 1% of the global population [1]. Disease-modifying antirheumatic drugs (DMARDs), including synthetic DMARDs, and biological DMARDs, are first-line therapies for RA. Several clinical trials have revealed remission rates of 50-80% in RA patients treated with DMARDs [2–5]; however, at least 20% do not respond to certain DMARD therapies, underscoring the importance of identifying predictive biomarkers, which could enable clinicians to provide more effective, early-stage therapies for RA.

The pathogenesis of RA is complex and involves both environmental and genetic factors [6]. Arachidonic acid (AA) derived eicosanoids, such as prostanoids and leukotrienes, have been shown to mediate RA progression [7, 8]. Their biosynthesis involves the release of AA from membrane glycerophospholipids by cytosolic phospholipase A_2_ (cPLA_2_), cyclooxygenation and hydroperoxidation into prostaglandin H_2_ (PGH_2_) by cyclooxygenases (COX), and hydroperoxidation and dehydration into leukotriene A_4_ (LTA_4_) by 5-lipoxygenase (5-LO) and 5-LO-activating protein (FLAP). As the levels of these lipids are low in blood and their measurements could be challenging and irreproducible due to many factors, analyzing their urinary metabolites presents a viable alternative reflecting the systemic biosynthesis over a longer period of time [9]. Levels of urinary leukotriene E4 (LTE_4_), 12-hydroxyeicosatetraenoic acid (12-HETE), and metabolites of prostaglandin E_2_ (PGE_2_), prostaglandin D_2_ (PGD_2_), prostacyclin (prostaglandin I_2_) and thromboxane B_2_ (TXB_2_) are increased in several inflammatory diseases [10]. Although urinary tetranor-prostaglandin D metabolite (tPGDM), TXB_2_ metabolite (11-dehydro TXB_2_), and LTE_4_ have been shown to be elevated in RA patients [11, 12], it is still unclear how these metabolites might be affected by treatments of DMARDs.

Previous study described higher levels of plasma 6-trans leukotriene B_4_ (6-trans LTB_4_), 6-trans-12-epi LTB_4_, LTE_4_, PGE_2_, PGD_2_, and TXB_2_ in RA patients unresponsive to DMARDs [13]. However, these findings are questionable due to methodological flaws and technical challenges in measuring these lipids in blood [9, 14]. In the present research, we aim to investigate the effects of DMARDs on urinary levels of tPGEM, tPGDM, 2,3-dinor-6-keto prostaglandin F_1α_ (PGIM), 2,3-dinor thromboxane B_2_ (TXBM), LTE_4_ and 12-HETE in RA patients. In addition, we aim to determine if baseline urinary eicosanoid levels differ between responders and non-responders to DMARDs therapy.

## Materials and methods

### The study population

This study included 152 Swedish patients enrolled in the NORD-STAR trial (registration number: NCT01491815) (Table 1) [15]. All patients included are females and are positive for rheumatoid factor (RF). All patients recruited in the NORD-STAR trial fulfilled the 2010 ACR/EULAR classification criteria and had symptom duration of less than 24 months. These participants, stratified by anti-citrullinated protein antibodies (ACPA) positivity, were evenly randomized into four groups, each receiving methotrexate (MTX) from day one alongside one of four DMARDs: (i) prednisolone (arm ACT), (ii) TNF-α blocker certolizumab pegol (arm CZP), (iii) CTLA-4Ig abatacept (arm ABA), or (iv) IL-6R blocker tocilizumab (arm TCZ). While intra-articular corticosteroid injections were permitted, they were not allowed within four weeks prior to sample collection. Clinical outcomes include clinical disease activity index (CDAI), C-reactive protein (CRP) and erythrocyte sedimentation rate (ESR). Clinical remission is defined as CDAI ≤ 2.8 [16]. Urine samples were collected at baseline and 24 weeks post-treatment, and stored at -80℃ for subsequent analysis. All patients recruited in the study were free from urinary tract infections within 1 month prior to urine sample collection.

**Table 1:** Characteristics of 152 Swedish female patients with early RA included in the study

	Prednisone and MTX (ACT arm)	Certolizumab Pegol and MTX (CZP arm)	Abatacept and MTX (ABA arm)	Tocilizumab and MTX (TCZ arm)	P
N	38	42	39	33	
Age	53.42 ± 16.74	51.93 ± 14.78	54.26 ± 16.19	53.39 ± 13.17	0.928^a^
BMI	26.11 ± 5.81	24.97 ± 4.79	26.18 ± 4.70	25.92 ± 5.25	0.690^a^
Current smoker (%)	15.79%	16.67%	10.26%	24.24%	0.464^b^
ACPA positive (%)	84.21%	85.71%	87.18%	84.85%	0.985^b^
**CDAI**					
Day 0	32.86 ± 14.51	30.53 ± 13.67	29.19 ± 19.00	27.68 ± 11.28	0.349^a^
Week 12	7.51 ± 6.04	5.79 ± 4.77	6.02 ± 5.62	8.09 ± 8.98	0.335^a^
Week 24	6.11 ± 5.69	4.25 ± 4.42	4.65 ± 5.43	6.23 ± 5.83	0.260^a^
Remission-w24^*^ (%)	36.84%	52.38%	51.28%	42.42%	0.461^b^
EULAR-Good response-w24 (%)	78.95%	88.1%	84.62%	69.7%	0.312^b^
**CRP**					
Day 0	20 (9.75–40.25)	13 (4.6–32)	8.8 (4–30)	8 (4.25-20)	0.078^c^
Week 24	2 (1-4.5)	1 (0.5–2.08)	2 (1-3.8)	0.5 (0.4-1)	**< 0.001** ^**c**^
**ESR**					
Day 0	35 (23.75–57.5)	32 (19–58)	28 (17–50)	26 (16.5-39.75)	0.273^c^
Week 24	11 (5.75-21)	9.5 (5.75–19.5)	11 (7–23)	2 (2–4)	**< 0.001** ^**c**^

Data was presented as mean ± SD for normally distributed variables and median (IQR) for variables with a skewed distribution

ACT, Active Conventional Therapy arm; CZP, Certolizumab Pegol arm; ABA, Abatacept arm; TCZ, Tocilizumab arm; MTX, Methotrexate; BMI, Body Mass Index; ACPA, Anticitrullinated peptide antibody; CDAI, Clinical Disease Activity Index; EULAR, European Alliance of Associations for Rheumatology; CRP, C Reactive Protein; ESR, Erythrocyte Sedimentation Rate

^a^One-way ANOVA

^b^Pearson Chi-square

^c^Kruskal–Wallis test

*Remission was defined as CDAI ≤ 2.8

### Lipid extraction and liquid chromatography–mass spectrometry (LC-MS)

Urine samples were thawed on ice or in a fridge (4^o^C) and centrifuged at 1,000 g for 5 min at 4℃ to remove any precipitant. Each 250 µl urine sample was spiked with 50 µl of an internal standard (IS) mixture containing 0.4 µg/ml of tPGEM-d_6_, tPGDM-d_6_, 2,3-dinor-6-keto PGF_1α_-d_9_, 2,3-dinor TXB_2_-d_9_, LTE_4_-d_5_ and 12-HETE-d_8_ (Cayman Chemicals, Michigan, United States). Samples were diluted with 700 µl of 10mM ammonium acetate (AmAc) at pH 9.5 (Merck, Darmstadt, Germany), and 5 µl of 5% NH_4_OH (Sigma Aldrich, Missouri, United States) was added to ensure a final pH at 10 ± 0.5. The prepared samples were then loaded to a pre-activated and equilibrated Oasis MAX 96-well plate (Waters, Massachusetts, United States), washed with 1 ml AmAc (10mM pH 9.5) and 1 ml 100% acetone (Merck, Darmstadt, Germany). Oxylipins were eventually eluted in 1 ml acetone containing 3% acetic acid, then evaporated with SpeedVac and reconstituted in 50 µl 10% methanol in Milli-Q water. All urine samples were extracted in duplicates.

Reconstituted samples were analyzed by liquid chromatography on a Waters ACQUITY UPLC I-Class PLUS System (Waters Corporation, Massachusetts, United States) coupled to a triple quadrupole mass spectrometer, Waters Xevo TQXS Mass Spectrometer (Waters Corporation, Massachusetts, United States). Separation was achieved on a Waters ACQUITY UPLC BEH C18 column (2.1*50 mm, 1.7 μm) with a guard cartridge (Waters Corporation, Massachusetts, United States). The temperature of the analytical column was set to 60 °C and the flow rate was set to 0.5 ml/min. Mobile phase A was 0.05% formic acid in Milli-Q water, and mobile phase B was acetonitrile: isopropanol 9:1 with 0.05% formic acid. For LC-MS, two different methods were used in this study. Samples from arm CZP and arm TCZ were analyzed in the following gradient: initial 5% B, held at 5% B from 0.0 to 2.0 min, 5 to 10% B from 2.0 to 3.0 min, 10 to 25%B from 3.0 to 3.5 min, 25 to 35% B from 6.5 to 6.5 min, 35 to 95% B from 6.5 to 8.0 min, held at 95% B from 8.0 to 9.0 min, 95 to 5% B from 9.0 to 9.5 min and held 10%B from 9.5 to 11.0 min. After all samples were injected and analyzed for tPGEM and tPGDM, 10 µl methoxyamine hydrochloride (0.5 g/ml, Sigma-Aldrich, Missouri, United States) was added into the remaining samples and were incubated for 15 min at room temperature for full derivatization. Derivatized PGIM and TXBM were analyzed in the same gradient introduced above. Samples form arm ACT and ABA were analyzed in an optimized method, where tPGEM, tPGDM, LTE_4_ and 12-HETE were first analyzed in the following gradient: initial 5% B, held at 5% B from 0.0 to 1.5 min, 5 to 10% B from 1.5 to 2.5 min, 10 to 34%B from 2.5 to 4.0 min, 34 to 36% B from 4.0 to 8.5 min, 36 to 95% B from 8.5 to 14.0 min, held at 95% B from 14.0 to 15.0 min, 95 to 5% B from 15.0 to 15.5 min and held 10%B from 15.5 to 17.0 min. After derivatization, PGIM and TXBM were analyzed in a different gradient: initial 5% B, held at 5% B from 0.0 to 1.5 min, 10 to 35% B from 1.5 to 5.5 min, 35 to 95% B from 5.5 to 7.0 min, held at 95% B from 7.0 to 8.0 min, 95 to 5% B from 8.0 to 8.5 min and held 10% B from 8.5 to 11.0 min. Oxylipins were detected in multiple reaction monitoring (MRM) mode. The quantification transition, retention time (RT), lowest limit of detection (LLOD) and lowest limit of quantification can be found in Supplementary Table 1. Mass spectrometry data were analyzed with MassLynx (Version 4.20) software and quantifications were done using external standard curves with IS. In the present study, tPGEM and tPGDM were quantifiable in all samples. However, TXBM, PGIM, 12-HETE, and LTE4 were only quantifiable in some samples, with their positivity rather than absolute levels being analyzed. The levels of tPGEM and tPGDM were normalized to creatinine concentrations, which were measured by ELISA (#500,701, Cayman Chemicals, Michigan, United States) according to the manufacturer’s instructions. Total prostanoids were calculated by summing up all quantifiable prostanoids, including tPGEM, tPGDM, PGIM and TXBM, and normalized to creatinine concentrations.

### Statistical analysis

This study employed generalized estimating equation models to examine the impact of DMARDs on oxylipin production and to distinguish between responders (patients who achieved remission at week 24) and non-responders (patients with active disease at week 24). These models were adjusted for covariates including the use of nonsteroidal anti-inflammatory drugs (NSAIDs), creatinine concentrations, and baseline CDAI levels. Additionally, various statistical methods were used as appropriate and stated in the legends of each table/figure. These statical models include the student’s t-test, paired t-test, Pearson Chi-square test, Mann-Whitney U test, Spearman’s correlation test, repeated measures ANOVA, one-way ANOVA, and Kruskal–Wallis test. Statistical significance was determined at P values below 0.05. All analyses were conducted using SPSS Statistics software (IBM, New York, United States).

## Results

### Characteristics of the selected study population

We have selected 152 Swedish female patients who were RF positive across four treatment arms (Table 1). These patients exhibited comparable age, body mass index (BMI), smoking status, ACPA positivity, and baseline levels of CDAI, CRP, and ESR across the different arms, and seem to be representative for the NORD-STAR trial. When comparing the therapeutic effects between the treatments, we didn’t find any difference in CDAI at weeks 12 and 24, the percentage of patients achieving remission at week 24, or the percentage of patients considered to have a good response according to EULAR recommendations. However, a trend was noted indicating that patients treated with biological DMARDs were more likely, albeit not statistically significant, to achieve CDAI remission compared to those in the ACT arm. Notably, tocilizumab demonstrated a superior effect in reducing acute phase reactants compared to other drug combinations (Table 1). These findings align with those reported in the whole NORD-STAR trial [17], suggesting that our selection of patients represent the NORD-STAR cohort well.

### Urinary tPGEM, tPGDM, and TXBM were elevated specifically in patients who received anti-TNF and anti-IL6R treatments

To understand the effects of DMARDs on prostanoids production, we first studied the potential influence of NSAIDs uptake on prostanoid levels. Samples were considered NSAID-affected if NSAIDs had been used 2 weeks prior to sample collection. The number of NSAID-affected samples can be found in Supplementary Table 2. We found that NSAID-affected samples exhibited markedly lower levels of tPGEM and tPGDM, and were less likely to be tested positive for PGIM and TXBM (Supplementary Table 3). This highlights the necessarily to include “use of NSAIDs” as a covariate in the statistical model. As PGIM, TXBM, LTE_4_ and 12-HETE were not quantified in all samples and were analyzed based on their positivity, it is important to know if their detection is affected by urine concentration variances. We found that samples testing positive for these eicosanoids were significantly more concentrated than those testing negative, as evidenced by creatinine concentration comparisons (Supplementary Fig. 1A). Additionally, creatinine levels varied across samples from different therapeutic regimens and collection time points (Supplementary Fig. 1B), necessitating the inclusion of creatinine concentration as another covariate in the GEE models where PGIM, TXBM, LTE_4_ and 12-HETE were analyzed.

**Fig. 1:**
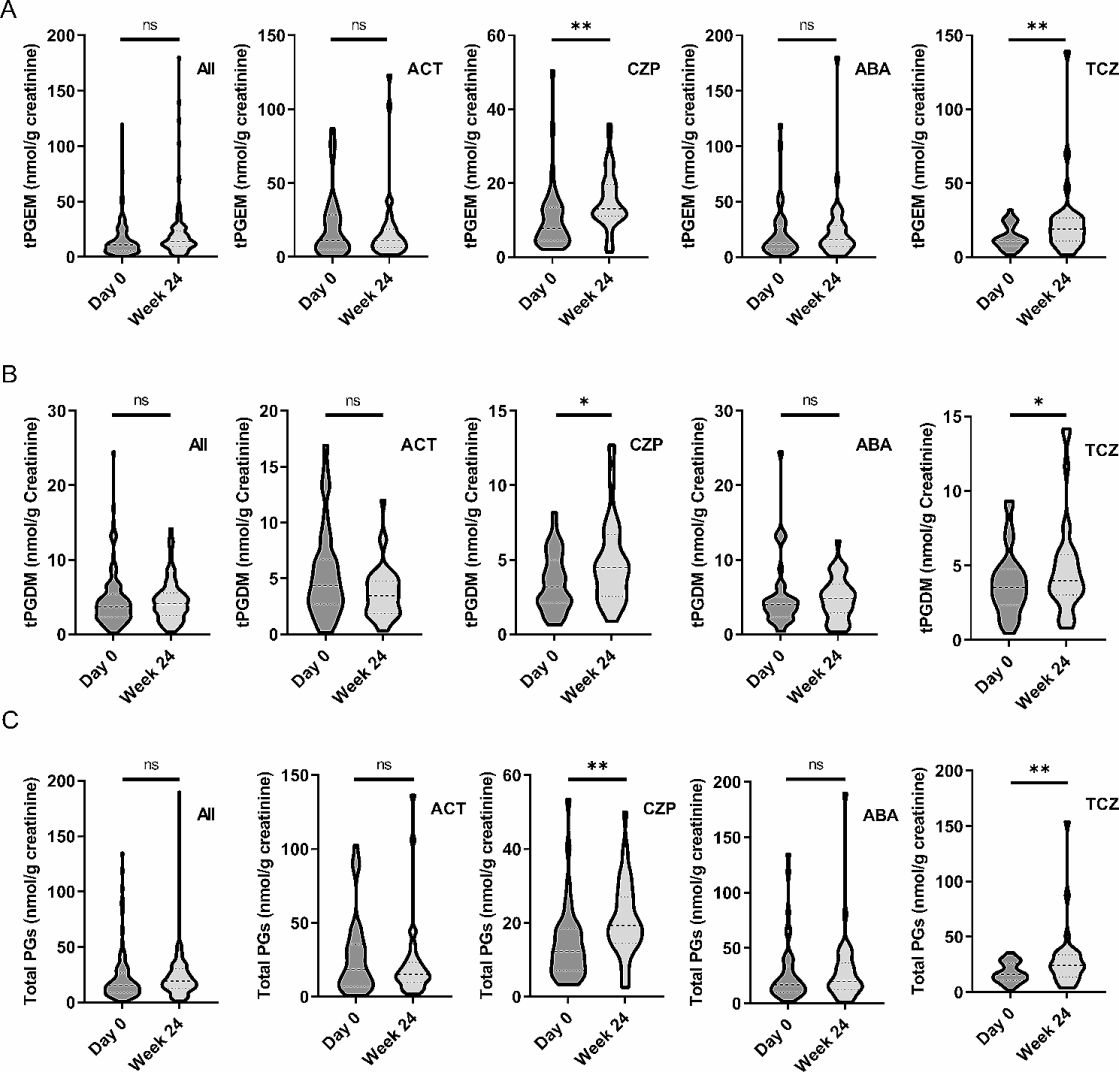
Effects of 4 therapeutic regimens on levels of tPGEM, tPGDM and total urinary prostanoids. Levels of tPGEM (**A**), tPGDM (**B**) and total urinary prostanoids (**C**) concentrations were measured in urine samples collected at baseline (Day 0) and week 24. Prostanoid concentrations were normalized to creatinine levels. Total prostanoids also include PGIM and TXBM when their levels are quantifiable (> LLOQ). Comparisons were performed across all patients and within individual arms. GEE models were used and use of NSAIDs were adjusted in the model. *P < 0.05, **P < 0.01. ACT, Active Conventional Therapy arm; CZP, Certolizumab Pegol arm; ABA, Abatacept arm; TCZ, Tocilizumab arm; GEE, Generalized Estimating Equations; NSAIDs, Non-Steroidal Anti-Inflammatory Drugs

To determine the effects of DMARDs on urine eicosanoid production, we first compared the levels of eicosanoids at baseline and 24 weeks post-treatment in 152 patients from all therapeutic arms. We didn’t find any changes in tPGEM, tPGDM and total prostanoid, or positivity for PGIM, TXBM, LTE_4_ and 12-HETE (Fig. 1; Table 2). As CZP and TCZ are known to target upstream signal pathways of eicosanoid biosynthesis, we further analyzed the effects of different treatment combinations separately. Surprisingly, we observed significant increase, instead of decrease, in tPGEM, tPGDM and total prostanoid among patients in arm CZP and TCZ (Fig. 1). Correspondingly, the proportion of TXBM-positive samples notably increased in these two arms (Table 2). Despite PGIM positivity did not significantly rise in these two arms, there’s a trend of increase. To unequivocally rule out any potential interference from NSAIDs, we conducted a parallel analysis on patients who had not consumed any NSAIDs during the initial 24 weeks. This analysis confirmed that increased production of urinary tPGEM and TXBM was exclusive to patients treated with CZP and TCZ (see Supplementary Table 4). Collectively, these results indicate that COX derived AA metabolites are increased in patients treated with anti-TNF and anti-IL6 drugs.

**Table 2:** Impacts of 24 weeks therapies of DMARDs on urinary PGIM, TXBM, 12-HETE and LTE_4_

	PGIM			TXBM			12-HETE			LTE_4_		
	Day 0	Week 24	P^a^	Day 0	Week 24	P^a^	Day 0	Week 24	P^b^	Day 0	Week 24	P^b^
Total	25.66%	40.79%	0.069	43.42%	69.74%	0.056	67.53.%	72.73%	0.419	23.38%	28.57%	0.669
ACT	28.95%	50.00%	0.172	65.79%	76.32%	0.723	63.16%	78.95%	0.098	23.68%	26.32%	0.904
CZP	19.05%	30.95%	0.320	16.67%	61.90%	**< 0.001**	Not measured					
ABA	38.46%	51.28%	0.194	66.67%	82.05%	0.131	71.79%	66.67%	0.653	23.08%	30.77%	0.588
TCZ	15.15%	30.30%	0.218	24.24%	57.58%	**0.019**	Not measured					

ACT, Active Conventional Therapy arm; CZP, Certolizumab Pegol arm; ABA, Abatacept arm; TCZ, Tocilizumab arm; PGIM, 2,3-dinor-6-keto-PGF_1α_; TXBM, 2,3-dinor-TXB_2_, 12-HETE, 12-Hydroxyeicosatetraenoic Acid; LTE_4_, Leukotriene E_4_; GEE, Generalized Estimating Equations

P values were obtained from the following statistical tests:

^a^GEE model adjusted for use of NSAIDs and creatinine concentrations

^b^GEE model adjusted for creatinine concentrations

### Baseline levels of urinary eicosanoids are not associated with baseline CDAI, and are not predictive of CDAI reduction or CDAI remission

Age of diagnosis, ACPA positivity and smoking status have been shown to be independent prognostic markers in RA [18–20]. Therefore, we first determined the association between levels of urinary eicosanoids and age/ACPA/smoking. Our analysis revealed no correlation between urinary eicosanoids and either age, ACPA positivity, or smoking (Supplementary Table 5). We next explored the correlation between levels of urinary eicosanoids and disease severity, and found no association between any eicosanoid and CDAI (Table 3). In addition, baseline levels of urinary eicosanoids did not correlate with a reduction in CDAI, nor did they vary between responders and non-responders (Table 3). Given the influence of muscle mass on creatinine production, which was used to adjust urine concentrations, we conducted further analysis incorporating BMI as an additional covariate. These analyses reaffirmed the absence of a correlation between baseline urinary eicosanoid levels and patient responses to treatment (Table 3). Considering that different DMARDs affect urinary prostanoids differently, we conducted the same correlation analysis within the separate treatment arms. This analysis also revealed no correlation with baseline CDAI, reduction in CDAI, or remission status at week 24 (Supplementary Table 6). In summary, our data do not support the use of urinary eicosanoids as biomarkers for treatment response of the analyzed drugs in early RA.

**Table 3:** Correlation between baseline levels of urinary eicosanoids and clinical outcomes

	CDAI Day 0				CDAI reduction (Day 0-Week 24) ^*^				Remission at week 24 (CDAI ≤ 2.8)^*^			
	Model 1		Model 2^§^		Model 1		Model 2^§^		Model 1		Model 2^§^	
	B	P value	B	P value	B	P value	B	P value	B	P value	B	P value
tPGEM^1^	-0.06	0.186	-0.06	0.240	-0.01	0.281	-0.02	0.394	-0.01	0.114	0.02	0.147
tPGDM^1^	0.05	0.834	0.01	0.980	0.01	0.835	0.07	0.591	-0.03	0.346	0.04	0.506
PGIM^2^	0.81	0.726	3.86	0.157	0.04	0.491	1.22	0.373	0.13	0.604	-0.13	0.809
TXBM^2^	0.46	0.826	2.09	0.477	0.01	0.849	1.02	0.311	-0.19	0.389	0.44	0.417
Total PGs^1^	0.04	0.274	-0.04	0.307	0.01	0.363	-0.01	0.515	-0.01	0.125	0.01	0.165
12-HETE^3#^	0.11	0.263	3.59	0.271	-0.09	0.268	-0.59	0.629	-0.09	0.863	0.09	0.869
LTE_4_^3#^	0.03	0.785	-0.78	0.777	0.01	0.912	2.04	0.255	0.21	0.733	-0.18	0.783

Generalized estimating equations (GEE) were used to calculate P values, adjustments were made as follows:

ACT, Active Conventional Therapy arm; ABA, Abatacept arm; CDAI, Clinical Disease Activity Index; PGIM, 2,3-dinor-6-keto-PGF_1α_; TXBM, 2,3-dinor-TXB_2_;12-HETE, 12-Hydroxyeicosatetraenoic Acid; LTE_4_, Leukotriene E_4_; NSAIDs, Non-Steroidal Anti-Inflammatory Drugs; BMI, Body Mass Index

^1^Adjust for NSAIDs

^2^Adjust for NSAIDs and creatinine concentrations

^3^Adjust for creatinine concentrations

*Adjust for CDAI day0

^§^Adjust for BMI at baseline

^#^Only measured in ACT and ABA arm

## Discussion

RA synovial tissues are known to express high levels of COX-2 and 5-LO [21–23]. Consequently, higher levels of AA metabolites derived from these oxygenase pathways have been detected in RA synovial fluid [8, 24]. Studies in animal models of inflammatory arthritis revealed significant pro-inflammatory and arthritogenic effects of PGE_2_/EP4 and LTB_4_/BLT1 signaling pathways [25–27]. Additionally, thromboxane and prostacyclin are also potent mediators of inflammation, and they might contribute to the cardiovascular comorbidity and pain behaviors in RA [28, 29]. In contrast, PGD_2_ and its metabolite 15-deoxy-∆^12,14^-prostaglandin J_2_ (15dPGJ_2_) exhibit anti-inflammatory effects in animal models of inflammatory arthritis [30, 31]. Therefore, these pro-inflammatory and anti-inflammatory lipid mediators derived from AA are considered as potential therapeutic targets and prognostic biomarkers in RA. NSAIDs, which inhibit inflammation by suppressing COXs, have been prescribed to RA patients since 1930s and remain in widespread use [32]. In our selection of patients, nearly 60% had used NSAIDs during the first 24 weeks of the trial (Supplementary Table 2). However, despite their efficient analgesic actions, NSAIDs can’t prevent or delay bone destructions themselves and may raise a series of gastrointestinal and cardiovascular side effects [33]. The adverse effects of NSAIDs are caused by disrupted TXA_2_/PGI_2_ balance, and might be avoided by using more selective inhibitors of PGE_2_ synthase, which might not only reduce production of pro-inflammatory PGE_2_, but also promote shunting to anti-inflammatory prostaglandins [34]. However, this therapeutic approach is not yet clinically available.

The potential of prostanoids and leukotrienes as prognostic biomarkers is understudied in RA patients. To our knowledge, only a single study has investigated the potential of plasma eicosanoids in predicting the response to DMARDs [13]. This study identified significantly higher levels of various eicosanoids derived from AA, including PGE_2_, PGF_2α_, PGD_2_, TXB_2_, LTB_4_, 6-trans LTB_4_, 6-trans-12-epi LTB_4_, LTE_4_, 5,12-diHETE and 5,15-diHETE, in the plasma collected from patients who didn’t respond to DMARDs [13]. However, analyzing eicosanoids in blood samples could be unreliable due to many reasons. Firstly, levels of eicosanoids in blood are notably low (usually less than 10 pg/ml) and unstable due to β- and ω-oxidations. More importantly, both prostanoids and leukotrienes can be formed ex vivo during blood collection, potentially leading to measured concentrations that are over tenfold higher than in samples collected with COX and 5-LO inhibitors [35]. Eicosanoid metabolites in the urine, on the contrary, are much more stable and offer more reproducible results [9, 35]. Urinary eicosanoids could be both excreted from the kidney and formed by the kidney. It is usually assumed that the more intermediate metabolites (ex. 6-keto PGF_1α_) are synthesized locally and the end products (ex. 2,3-dinor-6-keto PGF_1α_) reflect systemic production of the body [10]. Despite that the secretion of these eicosanoids might be affected under extreme kidney failure conditions, measuring urinary eicosanoids still represents the most reliable approach to study their systemic production. To this end, we measured urinary β-oxidation metabolites of PGE_2_, PGD_2_, thromboxane, and prostacyclin in RA patients and found no differences between those who responded to DMARDs and those who didn’t (Table 3 and Supplementary Table 6). Our data clearly negate the predictive values of these urinary oxylipins metabolites in RA. However, it should be noted that eicosanoids exert their biological effects in a localized manner, meaning that the systemic production of eicosanoids measured in the urine or blood might not be relevant to joint inflammation in RA. This is further evidenced by the lack of associations between urinary eicosanoids and clinical disease activity index (Table 3 and Supplementary Table 6), or CRP and ESR (data not shown). Therefore, the potential of eicosanoids in synovial fluid as predictors of DMARD response in RA patients warrants further investigation.

Expression of COX-2 is up-regulated in RA synovial tissue as a result of surrounding pro-inflammatory environment [8]. Pro-inflammatory cytokines, such as TNF-α and IL-6, induce expression of COX-2 in synovial fibroblasts and macrophages [36–38]. Therefore, anti-TNF and anti-IL6 treatments are expected to reduce production of COX-2 derived prostanoids. Contrary to this expectation, we observed an increase in urinary prostanoids in RA patients treated with CZP and TCZ (Fig. 1; Table 2). Notably, this elevation was not present in patients in arm ACT and ABA, suggesting that the increase is a specific effect of anti-TNF and anti-IL6 treatments rather than MTX. This finding aligns partially with our previous observations where TNF blockers, such as etanercept and infliximab, did not reduce COX-2 expression in RA synovial tissue, despite their significant inhibitory effects in vitro [23]. However, the mechanisms underlying the increase in prostanoid production by anti-TNF and anti-IL6 therapies remain unclear.

The presented research has several limitations: (i) Our study exclusively included seropositive female patients, necessitating validation of our results in a broader RA population; (ii) The sensitivity of our analytical protocol was insufficient to quantify TXBM, PGIM, LTE_4_ and 12-HETE in all samples, which hinders our ability to accurately assess changes in these eicosanoids. In addition, we analyzed our samples in two separate batches, with arm CZP and TCZ analyzed in the first batch, and arm ACT and ABA in second. Some samples were analyzed in both batches to exclude any batch effects. However, we employed different LC-MS methods for TXBM and PGIM across these two batches. After the first batch, we optimized the method to enhance sensitivity, leading to differences in baseline positivity rates for various regimens (Table 2). To ensure the validity of our conclusion that TXBM and PGIM positivity did not increase in the ACT and ABA arms, we redefined ‘positive’ with the LLOQ of the first batch method (80 fmol for TXBM and PGIM) in the data from the second batch. This adjustment yielded consistent results, indicating no significant differences in TXBM and PGIM positivity between baseline and 24 weeks post-treatment (data not shown). Nonetheless, the use of different analytical methods across batches may limit the direct comparability of data between different treatment arms.

## Conclusions

To the best of our knowledge, we are the first to study the effects of various DMARDs on urinary eicosanoids production in RA patients. We discovered that patients treated with certolizumab pegol and tocilizumab had significantly increased production of urinary prostanoids after 24 weeks of therapies. However, baseline levels of eicosanoids are not associated with clinical disease activity or response to treatments.

### Electronic supplementary material

Below is the link to the electronic supplementary material.

Supplementary Material 1

## Data Availability

The data underlying this article will be shared on reasonable request to the corresponding author (Per-Johan Jakobsson).

## Abbreviations

15dPGJ_2_: 15: deoxy-∆12,14-prostaglandin J2
AA: Arachidonic Acid
ABA: Abatacept
ACPA: Anti-Citrullinated Protein Antibodies
ACR: American College of Rheumatology
ACT: Active Conventional Treatment
AmAc: Ammonium Acetate
BMI: Body Mass Index
CDAI: Clinical Disease Activity Index
cPLA_2_: cytosolic phospholipase A2
COX: Cyclooxygenases
CRP: C-Reactive Protein
CZP: Certolizumab Pegol
DMARDs: Disease-Modifying Antirheumatic Drugs
ESR: Erythrocyte Sedimentation Rate
EULAR: European League Against Rheumatism
GEE: Generalized Estimating Equation
HETE: Hydroxyeicosatetraenoic acid
IL6: Interleukin 6
IS: Internal Standard
LC-MS: Liquid Chromatography–Mass Spectrometry
LLOD: owest Limit of Detection
LLOQ: Lowest Limit of Quantification
LO: Lipoxygenase
LTA_4_: Leukotriene A4
LTB_4_: Leukotriene B4
LTE_4_: Leukotriene E4
MO: Methoxyamine
MRM: Multiple Reaction Monitoring
MTX: Methotrexate
NORD-STAR: Nordic Rheumatic Diseases Strategy Trials and Registries
NSAIDs: Nonsteroidal Anti-Inflammatory Drugs
PGD_2_: Prostaglandin D2
PGE_2_: Prostaglandin E2Prostaglandin E2
PGH_2_: Prostaglandin H2
PGIM: 2,3-dinor-6-keto prostaglandin F1α
PGI_2_: Prostaglandin I2, prostacyclin
RF: Rheumatoid Factor
RT: Retention Time
TCZ: Tocilizumab
TNF-α: Tumor Necrosis Factor-α
tPGDM: tetranor-Prostaglandin D metabolite
tPGEM: tetranor-Prostaglandin E metabolite
TXB_2_: Thromboxane B2
TXBM: 2,3-dinor thromboxane B2
