# Supplementary material for: Urinary prostanoids are elevated by anti-TNF and anti-IL6 receptor disease-modifying antirheumatic drugs but are not predictive of response to treatment in early rheumatoid arthritis
Source: Arthritis Res Ther. 2024 Mar 5;26:61. doi: 10.1186/s13075-024-03295-9 (PMC10913231; doi:10.1186/s13075-024-03295-9)
Supplement: Supplementary file 1 — Supplementary Material 1 [file 13075_2024_3295_MOESM1_ESM.docx]

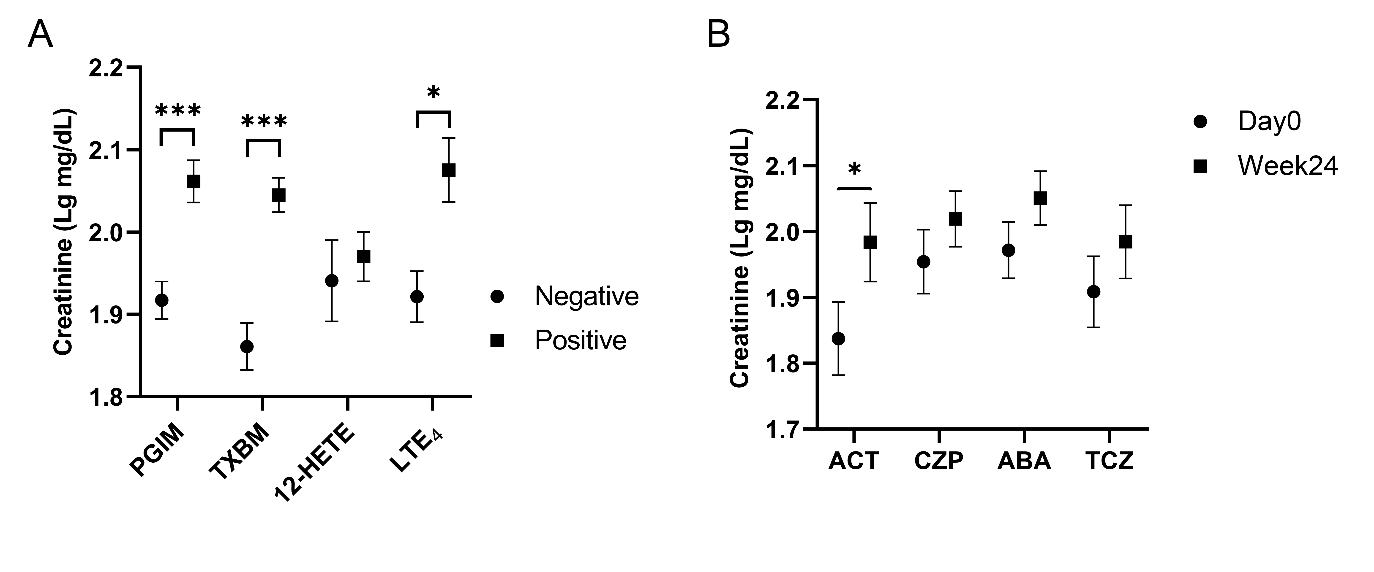


Supplementary Figure 1: Creatinine levels are different between sample groups

(A) Creatinine levels between samples tested positive and negative for urinary eicosanoids. Student's t-tests were used for statistical analysis.

(B) Creatinine levels between samples collected at baseline and 24 weeks post-therapy in individual arms. Paired t tests were used for statistical analysis.

*P<0.05, ***P<0.001.

Supplementary Table 1: MRM methods for urinary eicosanoids

|  | **Parent Ion (m/z)** | **Daughter Ion (m/z)** | **RT (min)** | **LLOD (injected to column)** | **LLOQ (injected to column)** |
| --- | --- | --- | --- | --- | --- |
| Method used for samples from arm CZP and TCZ | | | | | |
| tPGEM | 327 | 309 | 3.33 | Not determined | 23 fmol |
| tPGDM | 327 | 309 | 3.53 |  | 11 fmol |
| PGIM | 370 | 150 | 4.95 |  | 80 fmol |
| TXBM | 370 | 155 | 5.18 |  | 80 fmol |
| Optimized method used for samples from arm ACT and ABA | | | | | |
| tPGEM | 327 | 309.2 | 2.90 | 2.9 fmol | 23 fmol |
| tPGDM | 327 | 309.2 | 3.07 | 1.5 fmol | 6 fmol |
| PGIM | 370.3 | 150.1 | 5.32 | 1.5 fmol | 6 fmol |
| TXBM | 370.3 | 155.1 | 5.44 | 1.5 fmol | 6 fmol |
| 12-HETE | 319.5 | 179.1 | 11.64 | 2.9 fmol | 46 fmol |
| LTE_4_ | 438.2 | 333.2 | 9.41 | 5.8 fmol | 94 fmol |

In the optimized method, methoxyamine (MO) derivatized 2,3-dinor-TXB_2_ (TXBM) and 2,3-dinor-6-keto-PGF_1α_ (PGIM) were measured in a 10 minutes gradient while the others were measured in a 17 minutes gradient. The lowest limit of detection (LLOD) was defined as the peak with a signal to noise (S/N) above 3, and the lowest limit of quantification (LLOQ) was defined as the peak with a S/N above 10. 12-HETE and LTE_4_ were only measured in samples from patients in ACT and ABA arms.

Supplementary Table 2: Use of non-steroidal anti-inflammatory drugs (NSAIDs) in the study population

| **Arms** | **Day 0 only** | **W 24 Only** | **Both** | **None** |
| --- | --- | --- | --- | --- |
| ACT (N=38) | 12 (31.58%) | 0 (0.00%) | 10 (26.32%) | 16 (42.11%) |
| CZP (N=42) | 12 (28.57%) | 3 (7.14%) | 15 (35.71%) | 12 (28.57%) |
| ABA (N=39) | 11 (28.21%) | 0 (0.00%) | 4 (10.26%) | 24 (61.54%) |
| TCZ (N=33) | 1 (3.03%) | 3 (9.09%) | 17 (51.52%) | 12 (36.36%) |
| P value | <0.001 |  |  |  |

Use of NSAIDs was defined based on medication history 2 weeks prior to sample collection. A list of NSAIDs include: valdecoxib, indometacin, Diklofenak, diclofenac in combination, piroxicam, meloxicam, ibuprofen, naproxen, ketoprofen, mefenamic acid, celecoxib, nabumetone, Dexibuprofen, Glukosamin, and Etoricoxib. P value was obtained from Pearson Chi-square test.

ACT, Active Conventional Therapy arm; CZP, Certolizumab Pegol arm; ABA, Abatacept arm; TCZ, Tocilizumab arm.

Supplementary Table 3: Use of NSAIDs affect levels of urinary prostanoids

|  |  | **tPGEM (nmol/g)** | **tPGDM (nmol/g)** | **PGIM** | **TXBM** |
| --- | --- | --- | --- | --- | --- |
|  |  | Median with IQR | | Positivity | |
| NSAIDs Neg. | N=167 | 13.88 (9.01-25.53) | 11.07(5.41-18.05) | 40.72% | 65.27% |
| NSAIDs Pos. | N=137 | 4.26 (2.65-5.77) | 3.38 (2.19-5.23) | 24.09% | 45.99% |
| P values |  | 0.004 ^a^ | 0.040 ^a^ | 0.002 ^b^ | 0.001 ^b^ |

NSAIDs positivity was defined based on medication history 2 weeks prior to sample collection. Data was presented as median (IQR) for tPGEM and tPGDM as they are not normally distributed.

a. Mann-Whitney U test

b. Pearson Chi-square test

NSAIDs, Non-Steroidal Anti-Inflammatory Drugs; PGIM, 2,3-dinor-6-keto-PGF_1α_; TXBM, 2,3-dinor-TXB_2_.

Supplementary Table 4: Effects of 4 therapeutic regimens on urinary eicosanoids among NSAIDs-free patients

|  | ACT (N=16) | | | CZP (N=12) | | | ABA (N=24) | | | TCZ (N=12) | | |
| --- | --- | --- | --- | --- | --- | --- | --- | --- | --- | --- | --- | --- |
|  | BL | Week 24 | P | BL | Week24 | P | BL | Week24 | P | BL | Week24 | P |
| tPGEM (nmol/g) | 18.15 (9.40-28.39) | 13.23 (7.50-23.69) | 0.860 | 12.02 (4.84-14.85) | 19.30 (13.04-28.45) | **0.041** | 10.57 (5.88-27.48) | 14.78 (11.23-23.72) | 0.433 | 14.01 (8.19-21.83) | 22.66 (17.39-29.71) | **0.036** |
| tPGDM (nmol/g) | 4.89 (3.39-7.59) | 3.97 (2.19-5.12) | 0.032 | 3.91 (3.06-5.34) | 4.70 (3.19-9.44) | 0.062 | 3.60 (2.02-4.94) | 5.31 (3.65-6.81) | 0.104 | 3.31 (2.33-6.22) | 4.53 (3.21-7.10) | 0.245 |
| PGIM (Positivity) | 43.75% | 68.75% | 0.289 | 33.33% | 33.66% | 0.285 | 37.5% | 58.33% | 0.229 | 16.67% | 33.33% | 0.313 |
| TXBM (Positivity) | 93.75% | 87.5% | 0.221 | 25% | 58.33% | **0.046** | 62.5% | 83.33% | 0.315 | 25% | 66.67% | **0.027** |
| 12-HETE (Positivity) | 62.5% | 75% | 0.383 | Not measured | | | 70.83% | 58.33% | 0.322 | Not measured | | |
| LTE4 (Positivity) | 37.5% | 25% | 0.195 |  |  |  | 33.33% | 33.33% | 0.937 |  |  |  |

Generalized estimating equations (GEE) were used to calculate P values, creatinine concentrations were adjusted as a covariate when analyzing PGIM, TXBM, 12-HETE and LTE_4_.

ACT, Active Conventional Therapy arm; CZP, Certolizumab Pegol arm; ABA, Abatacept arm; TCZ, Tocilizumab arm; PGIM, 2,3-dinor-6-keto-PGF1α; TXBM, 2,3-dinor-TXB2, 12-HETE, 12-Hydroxyeicosatetraenoic Acid; LTE4, Leukotriene E4; GEE, Generalized Estimating Equations.

Supplementary Table 5: Urinary eicosanoids at baseline are not associated with age, ACPA positivity and smoking status

|  | **tPGEM** | | **tPGDM** | | **PGIM** | | **TXBM** | |  | **12-HETE** | | **LTE_4_** | |
| --- | --- | --- | --- | --- | --- | --- | --- | --- | --- | --- | --- | --- | --- |
| **Age (N=152)** | ρ | P | ρ | P | B | P | B | P | **Age (N=77)** | B | P | B | P |
|  | 0.139 | 0.087 ^a^ | 0.167 | 0.051 ^a^ | 0.018 | 0.121 ^b^ | 0.002 | 0.854 ^b^ |  | -0.08 | 0.252 ^b^ | 0.132 | 0.064 ^b^ |
|  | | | | | | | | | | | | | |
| **ACPA** | **tPGEM (nmol/g)** | | **tPGDM (nmol/g)** | | **PGIM (positivity)** | | **TXBM (positivity)** | | **ACPA** | **12-HETE (positivity)** | | **LTE_4_ (positivity)** | |
| Positive (N=130) | 10.81  (5.25-18.64) | | 3.56  (2.21-5.30) | | 24% | | 41% | | Positive (N=66) | 72.73% | | 24.24% | |
| Negative (N=22) | 12.83  (6.90-27.81) | | 4.24  (2.43-6.03) | | 36% | | 59% | | Negative (N=11) | 66.67% | | 18.18% | |
| P-Value (ACPA^+^ vs. ACPA^-^) | 0.187 ^c^ | | 0.402 ^c^ | | 0.214 ^d^ | | 0.109 ^d^ | | P-Value (ACPA^+^ vs. ACPA^-^) | 0.691 ^d^ | | 0.193 ^d^ | |
|  | | | | | | | | | | | | | |
| **Smoking** | **tPGEM (nmol/g)** | | **tPGDM (nmol/g)** | | **PGIM (positivity)** | | **TXBM (positivity)** | | **Smoking** | **12-HETE (positivity)** | | **LTE_4_ (positivity)** | |
| Smoker (current) | 14.08 (4.73-26.61) | | 3.60 (1.57-9.65) | | 30% | | 70% | | Smoker (current) | 60% | | 30% | |
| Non-smoker (current) | 11.89 (5.57-27.79) | | 4.29 (2.79-6.32) | | 34.33% | | 65.67% | | Non-smoker (current) | 68.66% | | 22.39% | |
| P-Value (smoker vs. non-smoker) | 0.574 ^c^ | | 0.287 ^c^ | | 0.836 ^d^ | | 0.706 ^d^ | | P-Value (smoker vs. non-smoker) | 0.586 ^d^ | | 0.596 ^d^ | |

12-HETE and LTE_4_ were only measured in ACT and ABA arms. Data on tPGEM and tPGDM were expressed as median (IQR) as they are not normally distributed. P values were calculated with the following statistical methods:

a. Spearman's correlation

b. Generalized estimating equations (GEE) adjusted for creatinine concentrations

c. Mann-Whitney U test

d. Pearson Chi-square test

ACPA, Anticitrullinated peptide antibody; PGIM, 2,3-dinor-6-keto-PGF_1α_; TXBM, 2,3-dinor-TXB_2_, 12-HETE, 12-Hydroxyeicosatetraenoic Acid; LTE_4_, Leukotriene E_4_.

Supplementary Table 6: P values for correlations between baseline levels of urinary eicosanoids and clinical outcomes in individual arms (in relation to Table 3).

|  | **ACT (N=38)** | | | **CZP (N=42)** | | | **ABA (N=39)** | | | **TCZ (N=33)** | | |
| --- | --- | --- | --- | --- | --- | --- | --- | --- | --- | --- | --- | --- |
|  | **CDAI Day 0** | **CDAI reduction (Day 0-Week 24) ^*^** | **Remission at week 24 (CDAI≤2.8)^*^** | **CDAI Day 0** | **CDAI reduction (Day 0-Week 24) ^*^** | **Remission at week 24 (CDAI≤2.8)^*^** | **CDAI Day 0** | **CDAI reduction (Day 0-Week 24) ^*^** | **Remission at week 24 (CDAI≤2.8)^*^** | **CDAI Day 0** | **CDAI reduction (Day 0-Week 24) ^*^** | **Remission at week 24 (CDAI≤2.8)^*^** |
| tPGEM^1^ | 0.392 | 0.551 | 0.941 | 0.348 | 0.297 | 0.269 | 0.427 | 0.311 | 0.380 | 0.110 | 0.509 | 0.354 |
| tPGDM^1^ | 0.727 | 0.643 | 0.910 | 0.533 | 0.983 | 0.899 | 0.624 | 0.708 | 0.236 | 0.489 | 0.387 | 0.940 |
| PGIM^2^ | 0.206 | 0.968 | 0.729 | 0.399 | 0.472 | 0.276 | 0.390 | 0.186 | 0.647 | 0.879 | 0.342 | 0.909 |
| TXBM^2^ | 0.25 | 0.544 | 0.349 | 0.193 | 0.421 | 0.528 | 0.855 | 0.898 | 0.425 | 0.535 | 0.768 | 0.975 |
| Total PGs^1^ | 0.424 | 0.558 | 0.960 | 0.452 | 0.357 | 0.350 | 0.513 | 0.414 | 0.369 | 0.126 | 0.460 | 0.467 |
| 12-HETE^3^ | 0.172 | 0.268 | 0.175 | Not measured | | | 0.946 | 0.736 | 0.168 | Not measured | | |
| LTE_4_^3^ | 0.731 | 0.912 | 0.622 |  |  |  | 0.422 | 0.357 | 0.545 |  |  |  |

Generalized estimating equations (GEE) were used to calculate P values, adjustments were made as follows:

1. adjust for NSAIDs

2. adjust for NSAIDs and creatinine concentrations

3. adjust for creatinine concentrations

* adjust for CDAI day0

ACT, Active Conventional Therapy arm; CZP, Certolizumab Pegol arm; ABA, Abatacept arm; TCZ, Tocilizumab arm; CDAI, Clinical Disease Activity Index; PGIM, 2,3-dinor-6-keto-PGF_1α_; TXBM, 2,3-dinor-TXB_2_; 12-HETE, 12-Hydroxyeicosatetraenoic Acid; LTE_4_, Leukotriene E_4_; NSAIDs, Non-Steroidal Anti-Inflammatory Drugs.
